# New York Academy of Medicine—Section on Orthopedic Surgery

**Published:** 1891-04

**Authors:** Samuel Ketch

**Affiliations:** Chairman


### Society Proceedings.

### NEW YORK ACADEMY OF MEDICINE.—SECTION ON ORTHOPEDIC SURGERY.

SAMUEL KETCH, M. D., Chairman.

FEBRUARY MEETING.

Dr. Charles N. Dixon Jones, of Brooklyn, reported a case of Congenital (Double) Equino-Varus, with exsection of both tarsi, and exhibited casts and photographs, as well as the patient.

Kate M., 11 years old. A few weeks after her birth the feet had been tenotomized, and an apparatus worn ever since. On February 29th both feet were operated upon by Phelps's open incision, and forcible rectification. In November of the same year a wedge of bone was removed from the cuboid in both feet. She was then treated by water-glass and plaster splints until early in 1889, when she disappeared for several months. On her return there was found to be a considerable relapse, with inversion of the feet and fixation of the joints of the tarsus. On November 29, 1890, the operation of Mr. Davies Colleys for resection of the tarsus was performed on both feet. At the end of four weeks the feet were in good position, and the wounds were nearly healed. This case was a very intractable one, and was the only one out of a number of cases of club-foot in the author's experience where it was found necessary to resort to tarsal resection.

The second case was one of resection of the astragalo-scaphoid articulation for aggravated flat foot. The patient and photographs were exhibited.

K. K., 10 years of age. The deformity caused great suffering. On examination it was found that the inner side of the right foot in its whole length rested upon the ground. The astragalo-scaphoid joint formed a well-marked prominence. On the 1st of February Ogston's operation was performed. The plaster splint was continued for eight weeks. She now walked comfortably.

Dr. Jones also reported two cases of excision of the hip for tubercular ostitis.

Tillie C., a delicate girl of four years of age, had suffered from the disease for eight months. Owing to high temperature and great pain it was decided to operate. The diseased bone was removed by a free incision, which gave exit to several ounces of pus. The diseased acetabulum was thoroughly curetted, and an extension apparatus applied. After the operation the patient experienced marked relief, and the temperature remained normal. The first dressing was removed at the end of a week, and the subsequent ones were made about every four days, with the patient under an anesthetic, and the parts were thoroughly curetted. The patient is able now to run and jump without any apparatus, and there is only one inch shortening.

The second case of excision was that of Annie M., who had had a tubercular coxitis for about one year. She was three years old, had never walked, and the pain was sufficient to seriously interfere with sleep. There was a fluctuating swelling over the joint. On November 3d a similar operation to that just described was performed, and the wound was treated openly according to the method advocated by Mikulitz. Recovery was rapid and uninterrupted.

The author felt confident that the frequent erasions of the joint surfaces formed an important element in the termination of the tubercular process.

Dr. V. P. Gibney said that he assumed that in the first case the club foot was probably the result of poliomyelitis, the anterior and posterior tibial muscles being chiefly affected ; and that in the effort to bring down the heel flat-foot had been produced. He thought that a still further improvement would follow the division of the tendo Achillis. The case seemed to him to be a good illustration of the necessity of continuing the use of protective apparatus for some time after the separations, for the history stated that the patient while still wearing only a plaster or water-glass splint passed from observation for some time, and when next seen, the plaster had been discontinued and the case had relapsed. The child walked rather tenderly, and the ankles rolled outward. The left foot could not be brought quite up to ninety degrees, and he perceived in it indications of a probable relapse. In such an event, he would suggest that the astragalus be removed according to the method of Morton, of Philadelphia. Nothing is lost by the removal of this bone, because it is really subluxated forward, and the claim which has been made that after this operation, the malleoli rest upon the os calcis, is of no significance, as they rest there before the removal of the astragalus. He had been surprised at the ease with which he could reduce the deformity after getting rid of the astragalus.

Dr. Royal Whitman considered the result obtained in the case of flat-foot a good one, but he did not approve of this class of operations. In this instance, a child of ten years had been confined to bed for ten weeks. If the foot had been over-corrected, under ether, and placed in a plaster bandage for the same length of time, even without the use of any apparatus, the result should have been equally good, and with the help of the apparatus and exercise, a very much better result might have been obtained without any cutting operation. Such operations, in his opinion, were unscientific.

Dr. A. B. Judson remarked that the occurrence of flat-foot as a result of infantile paralysis was rather unusual; it more commonly resulted in equino-varus or calcaneo-valgus.

Dr. R. H. Sayre did not agree with Dr. Judson that equino-valgus was rare after poliomyelitis when the anterior tibial muscle happened to be the chief one involved. The child was unable at present to hold the foot in that position in which it was normally held by this muscle. Under these circumstances, there is but little doubt that the deformity will recur. He did not believe that there was any such thing as a relapsed club-foot; such cases were simply instances of imperfect cures, in which the patients had been unable to voluntarily retain the foot in its normal position. A tenotomy of the tendo Achillis with retention of the foot for a long time in a corrected position would have answered in this case without any operation, although he thought the result obtained was one of the best he had ever seen after an osteotomy for flat-foot. Unless the foot could be brought to an angle of about one hundred and twenty degrees, locomotion, except with a high sole, was imperfect; yet, in all the cases of removal of the astragalus which he had seen, the feint between the astragalus and the tibia prevented the foot going beyond the right angle, and on this account he considered it inferior to the other operations. Fitzgerald, of Melbourne, had advocated a method of procedure which might almost be said to consist in reducing the whole tarsus nearly to a pulp by a series of osteotomies, and then molding the foot into the desired position, and holding it there with a plaster bandage. His published results of operations on some very badly deformed feet certainly appear most excellent. Dr. Jones's exsection of the hip-joint had yielded a remarkably beautiful result, and certainly it was preferable to obtain a joint with such good motion than to endeavor as do many of the foreign surgeons to obtain anchylosis.

Dr. Jones said that he considered Dr. Gibney's criticism on his first case a very just one. As to the second case, it was difficult to describe the many difficulties that he had encountered, and he had come to feel that nothing short of the heroic method of Dr. Fitzgerald would ever make it a good foot.

The Chairman presented a case which he had first seen in 1887. The young man was then in his fifteenth year. The family history showed freedom from rheumatism and joint disease, but there was phthisis on the maternal side. Nearly two years before this time the right knee became swollen, and one year later the ankles also swelled, and shortly afterward the left knee became similarly affected. His general health had always been good, and no cause could be assigned for this condition. Examination showed the right knee to be the seat of a large, doughy swelling; there was no pain on motion, and the movements of the joint were only limited by the mechanical obstacle offered by the swelling itself, and this only in extreme flexion. There was no elevation of temperature, either general or local. By hypodermic puncture a perfectly clear, colorless, syrupy fluid was withdrawn. He was treated first by plaster bandages, and afterward by elastic compression, counter-irritation, and systematic massage of the joints. The progress of the case had been slow and variable up to a few months ago, but since then it had been uninterrupted. There was still some fluctuation and enlargement of the right side, but he expected that the patient would ultimately recover completely. The case had been diagnosticated as hydrarthrosis.

Dr. Gibney said that the case was interesting, both on account of its comparative rarity and the excellent result which had been obtained.

Dr. A. M. Phelps had been accustomed in many of these cases of effusion into the joints, to open the joint and wash it out with a

1-2000 solution of bichloride, and he considered that it not only shortened the period of treatment, but was a safe practice, and gave equally good results as the more common method of treatment. He had often treated dispensary cases by this method, and after being in plaster-of-Paris for some time, they had been discharged in three months' time with good result. It was not uncommon to find fibrinous material as well as serum in the joint, and the removal of this along with the serum was beneficial, and the bichloride irrigation tended to excite a healthy inflammation of the synovial membrane, which hastened the process of recovery. We had been led to believe that these tubercular joints were always purulent, but he had occasion to examine many such joints microscopically, and had found the tubercle bacilli frequently present where there was no suppuration in the joint.

The Chairman, in closing the discussion on this case, said that it would be difficult to obtain the consent of most private patients to such an operation in a case like this, where there was so little disability or discomfort, and he thought the operation not only somewhat dangerous in itself, but liable to result, in a tuberculous case, in a general infection of the system.

The Chairman also presented a man, thirty-six years of age, whom he had first seen two days before. He gave a good family history as regards phthisis, joint and spinal disease, and said that he had enjoyed fair health excepting several attacks of rheumatism, the first of which occurred at ten, and the second at fourteen years of age. The third attack was severe, and occurred ten years ago, and involved only the right ankle. There was no venereal history. Two and a half years ago he was exposed for eight hours at night to wet and cold, and this was followed by pain in the left hip, passing down the side of the leg to the knee, and then across to the small of the back to the right hip. After that, he noticed his joints becoming stiff, yet there had been no pain, only a feeling of soreness upon motion. Both hip-joints have very little motion, adduction only allowing of the internal malleoli being brought within about thirteen inches of each other. The arms and hands are quite free, but there is slight restriction to the movements of the jaws. The patient states that he has been examined under ether, and that while under the influence of the anesthetic the motion of the joints was increased.

Dr. R. H. Sayre said that the improvement which the patient had been instrumental in procuring, in his own case, by constant efforts during the past six months to move the joints, suggested an appropriate line of treatment. Slight daily motions of the joints should be made while the patient is immersed in a bath at a temperature of 110-115 degrees. Such massage was more successful when aided by these hot baths, or by hot fomentations to the joints. He recalled one patient whose joints were so generally stiffened that she had been lying around almost helpless for three years, who, as a result of this treatment, was now able to walk without a cane, and with the motions of the elbows and shoulders very much improved. Such results were by no means exceptional, and he would be quite hopeful of decidedly improving this man's condition in the same way. When the joint is inflamed and tender, massage may render the inflammation sufficiently severe to cause anchylosis, but this man had been free from pain for a long time.

Dr. Gibney heartily approved of the suggestions which had been made, but he nevertheless believed that Dr. Sayre had had a singularly fortunate experience, and that usually these cases were very disappointing.

Dr. Phelps said that if this were a case similar to the one exhibited in the museum as "the ossified man," the hips, vertebræ, and even the jaws would become anchylosed in spite of treatment.

The Chairman, in closing the discussion, said that he had seen a number of these cases, and his experience had been unfortunate. The case should be classified as a rheumatoid arthritis, and this disease terminates in anchylosis. There were times in the course of the affection when there would be temporary amelioration. He did not favor operative procedures in such cases, but he thought the patient might be benefited by a course of massage and baths at the Hot Springs.

Dr. R. H. Sayre read a paper on The Importance of Thorough Examination in Suspected Pott's Disease. He said that although in childhood the signs of Pott's disease are usually so marked as not to be confounded with other troubles, in adults, especially in females, there are times when the diagnosis is not clear. In some cases of uterine displacement and ovarian diseases, the reflex pains, the posture and gait, may simulate Pott's disease so closely as to be mistaken for it by competent observers. Several such cases had fallen under the writer's notice.

In the first case, which the author related, a lady, twenty-six years of age, had received an injury of the right hip, which was followed by severe pains in the back and lower extremities. These pains were worse at night, and were so severe that she consulted a prominent Philadelphia physician. He pronounced the case one of Pott's disease, and applied a leather corset. This made her worse, and there was loss of power in the arms and legs. The jacket was was then removed, and she was advised to rest in bed for two or three years, but this advice was not followed. Two prominent New York physicians made the same diagnosis, and various braces and finally plaster were applied without benefit. She was still wearing the plaster jacket when she first came to the author. She could then walk only with difficulty; she was bent forward, and every jar caused pain. There was rigidity of the spinal muscles, and she complained of the girdle sensation, and of pains in the lower part of the abdomen and down the thighs. The uterus was found to be retroverted, and bound down by adhesions. An Alexander's operation, followed by the use of a pessary, faradism, and gymnastics, restored her to health.

The second case had had a spinal posterior brace applied by a London surgeon for supposed spinal disease. She complained of pain in the back and lower part of the abdomen. The uterus was retroverted, and the ovary prolapsed, and treatment directed to the relief of these conditions soon brought about a cure.

The third patient had worn various kinds of apparatus, and an examination showed a very slight knuckle in the dorsal region, which was thought to be due to an exaggeration of the physiological curve from her habitual stooping position resulting from the abdominal pain which she suffered. Her retroversion was corrected with a pessary, and she has since been free from pain.

The last case reported in the paper was that of an anemic girl, with a marked stoop, and a projection in the lumbar spine, with pain in the back, abdomen, and legs. She gave a history of dysmenorrhea, and the uterus was found markedly anteflexed. Tonics and general faradism improved her, and she has been without any support for over a year, without increase of the symptoms of Pott's disease.

In summing up the subject, the writer said that the description of these cases showed that the mistakes in diagnosis had been made by men of large experience, and he had therefore thought it worth while to call attention to the fact that reflex pains from pelvic irritation might easily lead one astray in considering cases of supposed Pott's disease.

### PATHOLOGICAL DISLOCATION OF THE HIPS.

Dr. W. R. Townsend presented a specimen of this condition, which had been removed from an Italian girl, fourteen years of age. The head of the femur was very deeply eroded, and was dislocated on to the dorsum ilii. There was marked erosion of the pelvic bones, but no perforation of the pelvis.

Dr. Townsend also presented a specimen illustrating acute arthritis in an infant of eleven months. There was no known cause for the condition which had lasted for two weeks prior to admission. There was a large gluteal abscess and the movements of the hips were somewhat circumscribed. As there were evidences of septicemia, an operation was performed with a view of securing proper drainage. The child died of exhaustion, and at the autopsy it was found that although the drainage was excellent, and the granulations appeared healthy, the head of the bone was eroded, and the external sinus communicated with the joint capsule. The viscera were perfectly healthy.

Dr. Judson said that the specimen illustrating pathological dislocation of the hip recalled a discussion which took place a few years ago on the question of the possibility of this dislocation. Dr. March, of Albany, argued that Dupuytren, Astley Cooper, C. Bell, Brodie, Listen, Fergusson, Miller, Gibson, Carnochan, and a host of other authorities were wrong in considering spontaneous dislocation in hip disease as a frequent occurrence. He declared that, as purely the result of morbid action unaided by superadded violence, it seldom, or never, took place. He visited forty pathological museums in all parts of the world, and failed to find evidences of this lesion. His forcible article in the Transactions of the American Medical Association, 1853, excited great opposition, and Dr. Hayward, of Boston, in his Surgical Reports, 1855, said it would require more specimens than would fill forty, or forty thousand museums, to convince him that a certain specimen, which he described, was not the result of spontaneous dislocation.

Before this discussion, spontaneous dislocation was supposed to be a very common incident of hip disease, in spite of the doubts expressed by Baron Larrey, and the statement by Wickham, in 1833, that it is of very rare occurrence. That dislocation is very often simulated when not really present, is not generally conceded. Dr. Gibney showed a specimen to the Pathological Society in 1877, in which dislocation was simulated by an appearance due to the altered direction of the neck of the femur. But that it sometimes does occur, is clear enough from the fine specimen in Dr. Townsend's hands.

There is another pathological dislocation of the hip that is worth considering from an orthopedic standpoint, *i. e.*, that is thought to be produced by distension of the capsule in the synovitis following continued fevers, as set forth by Dr. Keen in the Fifth Toner Lecture in 1877. He had recently examined a convalescent from typhoid fever, in whom there was great impairment of motion, and a distended capsule. Osteitis was eliminated by the history of the case, and by the absence of atrophy and natal symmetry. The patient was warned against undue disturbance of the joint, and recovered without dislocation, and without any special treatment. The subject is practically important because it is generally believed that serious joint diseases not infrequently have their origin in fevers.

Dr. Gibney said that he would like to know whether Dr. Townsend thought the child might have been saved if the head of the bone had been excised. A number of years ago Dr. Yale read a paper on incision of the hip, before the Surgical Society, and among other conclusions he stated that the best antipyretic for septicemia was excision of the hip.

Dr. Townsend replied that there was marked septicemia present at the time he had operated and drained the abscess, so that he doubted if the result would have been different had he excised the head of the bone. He thought, however, that any earlier operation would have saved the child's life. He had recently seen in Bellevue Hospital a man suffering from aggravated septicemia due to absorption cellulitis of the leg, who was so ill that it was feared he would die on the table during the amputation of the thigh; yet, instead of this, the amputation was followed by a very rapid improvement in his general condition.

